# Does inbreeding affect personality traits?

**DOI:** 10.1002/ece3.5487

**Published:** 2019-09-14

**Authors:** Magdalena Herdegen‐Radwan

**Affiliations:** ^1^ Department of Behavioural Ecology, Faculty of Biology Adam Mickiewicz University Poznań Poland

**Keywords:** boldness, condition, guppy

## Abstract

The question of why variation is maintained in personality traits is an evolutionary puzzle. According to the condition‐dependence hypothesis, such traits depend on condition, which limits the behavioral choices available to individuals. Because condition is affected by many genes, it can effectively be manipulated by inbreeding, which exposes the effects of deleterious recessive mutations. Here, I compared two personality traits, boldness and tendency to explore, of male guppies (*Poecilia reticulata*) from first‐generation inbred and outbred treatments. Boldness in guppies is associated with increased sexual attractiveness and is thus expected to affect fitness. Therefore, I hypothesized that the personality traits would be negatively affected by inbreeding. However, the results indicated that inbred guppies did not differ in either personality trait from their outbred counterparts. This finding suggests that mechanisms other than condition dependence are maintaining personality variation in the guppy.

## Graphical abstract

I tested condition dependence of personality traits using guppy. I manipulated condition via inbreeding and compared boldness and exploration of outbred and first‐generation inbred guppies. The results do not support the hypothesis of condition dependence of personality traits.

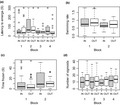

## 1 INTRODUCTION

In recent decades, reports have accumulated for a wide range of taxa on the presence of behaviors that differ within populations and/or among individuals but are individually stable. These behaviors are referred to as personality traits (Dingemanse & Reale, 2005; Gosling, 2001; Groothuis & Carere, 2005) and are characterized by consistency through time and context. Personality traits are important as they may influence within‐ and between‐species interactions, as well as have practical implications for research methodology. From an evolutionary perspective, the major question which remains unanswered is why do animals differ in personality, and how is the observed variation in personalities maintained? The question is particularly relevant given increasing evidence that personalities are linked to fitness (e.g., Patterson & Schulte‐Hostedde, 2011; Germano, Nafus, Perry, Hall, & Swaisgood, 2017; reviewed in Smith & Blumstein, 2008).

According to one of the proposed hypotheses, assuming the balancing selection scenario, none of the personalities is consistently selectively inferior. For example, Dingemanse, Both, Drent, and Tinbergen (2004) showed a complex pattern of selective pressures shaping exploratory behaviors, which act differently on gender and age class. They fluctuate in time with respect to food availability, thus promoting different levels of exploration depending on age and gender. Less attention has been devoted to a second scenario, which assumes that high‐fitness personalities are only available to individuals in good condition, whereas poor condition individuals are constrained to express low‐fitness personalities (Lewis, 2015; Luttbeg & Sih, 2010; Rands, Cowlishaw, Pettifor, Rowcliffe, & Johnstone, 2003). For example, bold individuals may often be at a higher risk of predation, or explorative ones may need more energy to maintain high activity. As a consequence, individuals in good condition, that is, having more resources available, may express more optimal levels of traits, while those in poor condition will not be able to afford it.

However, studies examining associations between personality traits and condition are scarce and their results contradictory (Kluen, Siitari, & Brommer, 2014; Kurvers, Adamczyk, Wieren, & Prins, 2011). This could at least partly be due to the difficulties inherent in measuring condition. In practice, the task is not straightforward, as no universally accepted phenotypic measure of condition exists (see e.g., Tomkins, Radwan, Kotiaho, & Tregenza, 2004), and single traits used as proxies of condition may be negatively genetically correlated with other fitness‐related traits (Stearns, 1992; Tomkins et al., 2004). For this reason, manipulating an individual's condition has been recommended as a preferred way to test condition dependence, and inbreeding offers an effective way to perform such a manipulation (see e.g., Bolund, Martin, Kempenaers, & Forstmeier, 2010). In inbred individuals, recessive deleterious mutations are exposed due to higher rates of average homozygosity (Charlesworth & Willis, 2009). Thus, condition, which is affected by many genes distributed throughout the genome (Rowe & Houle, 1996), is expected to deteriorate, a phenomenon called inbreeding depression (Charlesworth & Willis, 2009). Any trait linked to condition should also be prone to inbreeding depression, that is, a decrease in mean trait values under inbreeding (Tomkins et al., 2004, Prokop, Leś, Banaś, Koteja, & Radwan, 2010 and references therein, Simmons, 2011). Indeed, traits important for fitness, for example, life‐history traits, have been shown to be susceptible to inbreeding (DeRose & Roff, 1999).

An added value of manipulating condition via inbreeding (rather than e.g., by diet) is that within‐population variance in inbreeding may be an important source of variance in condition in natural populations. Indeed, Verweij et al. (2012) reported small yet significant associations between several personality traits in humans and the level of inbreeding estimated from the length of runs of homozygosity within the genome. Experimental inbreeding thus offers a valuable, but yet underused tool to explore reasons for the maintenance of personalities.

Here, I tested the relationship between inbreeding and personality traits using guppies (*Poecillia reticulata*), a small live‐bearing tropical fish from the family Poeciliidae, which is a model organism in evolutionary biology (Croft et al., 2011; Endler, 1983; Lucon‐Xiccato & Dadda, 2017; Reznick, Ghalambor, & Crooks, 2008). I reared the first generation of inbred and outbred male guppies in a common garden environment in order to compare two personality traits, boldness and exploration. Both traits have been shown to be associated with fitness components in this species: Smith and Blumstein (2010) found that bolder and more exploratory guppies survived longer when exposed to a predator, and Godin and Dugatkin (1996) discovered that female guppies preferred bolder males. Furthermore, in the population studied here, under laboratory conditions, I found a positive association between males' boldness and their competitive reproductive success (Herdegen‐Radwan, *in preparation*). Thus, these personality traits seem to be important components of individual fitness and good candidates for being condition‐dependent.

If variation in guppies' personality traits is maintained due to their condition dependence, I predicted personality traits to be negatively affected by inbreeding. This is because inbreeding is expected to deteriorate guppies' overall condition due to the exposure of slightly deleterious recessive mutations in many genes. This would result in a shift in mean population trait values toward lower fitness. Based on previous studies (Godin & Dugatkin, 1996, Herdegen‐Radwan *in preparation*), I expected that inbred guppies would be shyer and less explorative than their outbred counterparts. Courtship behavior, in which inbreeding depression has previously been demonstrated (Mariette, Kelley, Brooks, & Evans, 2006; van Oosterhout et al., 2003), and which was shown to be condition‐dependent (Nicoletto, 1993), was measured as a control for the effectiveness of the inbreeding treatment.

## 2 METHODS

### 2.1 Ethical note

The experimental protocols were approved by the 1st Local Ethics Committee in Poznań (decision numbers 5/2015 from 14.4.2015 and 69/2017 from 19.01.2018). The breeding population is licensed and monitored by the veterinary inspectorate (license no PL30646224 of the Local Veterinary Inspectorate in Poznań, Poland).

After the experiments, all inbred experimental fish were kept under the standard conditions described above, in unisex groups, until their natural deaths. They were not reused in any further experiments. Outbred fish were returned to the stock population.

### 2.2 Study population

Experimental fish came from a laboratory population established in 2010. They are descendants of Trinidadian guppies collected from Lower Tacarigua river (national grid reference PS 787 804) by Andrea Pilastro (University of Padova, Italy) in 2002. The laboratory population is bred in several 100 L aquaria with periodical exchange of a fraction of the fish between aquaria. There is still a high level of genetic variation maintained (Gasparini, Congiu, & Pilastro, 2015, own unpublished data). Fish within the stock and throughout the experiment were kept in stable conditions. These included a fixed temperature around 25 ± 1°C, an alternating light/dark regime every 12 hr and a feed twice per day (once with commercial dry flakes and once with nauplii of *Artemia* sp.).

### 2.3 Experimental design

Families were created by pairwise mating of mature males and virgin females from the stock population. Each pair occupied a 3‐L tank in a ZebTEC machine (Tecniplast®) which allowed for identical conditions in all experimental replicates. After one week, males were removed and females put into breeding chambers at the first signs of pregnancy. After giving birth, females were removed. To minimize environmentally induced differences in growth rate and maturity time, F1 fish from all families were kept in similar densities. As soon as males and females could be accurately distinguished, fish from each family were separated by sex and left undisturbed until maturity. At maturity, F1 families were randomly assigned to the inbred or outbred treatment. In the inbred treatment, one random male was paired with a random sister (“inbred pairs” from now on). In the outbred treatment, a random male from one family was paired with a random female from another family (“outbred pairs” from now on).

The procedure was repeated on these inbred and outbred pairs. They mated, females gave birth, and offspring were left to mature. At maturity, an F2 male from each family was chosen for behavioral tests and other measurements (see below). In order to avoid choosing the boldest or most conspicuous of the males, all brothers were first caught into a net, and then, one of them was picked without looking closely. In female guppies, intra‐individual variation in behavior has been observed which can be attributed to their ovarian cycle (Warren & Callaghan, 1975). Due to this variation, only males were used to assess personality traits in order to avoid such a confounding factor, as well as the confounding effect of sex. All tests and measurements were carried out blindly with respect to treatment.

I created a total of 173 experimental families over the course of four independent blocks. The number of males from each block and procedure used for the analyses are given in Table 1. Emergence test trials and sigmoid display observations were carried out in all four blocks, whereas for logistic reasons, open‐field tests were conducted in blocks 1 and 2. All tests were recorded for posttrial analysis with a Microsoft LifeCam Studio camera allocated above the test arena.

**Table 1:** Number of males (one from each family) that underwent behavioral tests, reported separately for each block and treatment, and jointly (grand total in bold)

Block	Inbred	Outbred	Total per block
1	25	26	51
2	29	25	54
3	16	22	38
4	13	17	30
Total per treatment	83	90	**173**

To get an estimate of the repeatability of personality tests, they were repeated in one‐week interval, similar to Burns (2008), in block 1. Courtship observation repeatability was estimated in block 3, where the second trial was carried out after 10 weeks, a time interval similar to that used by Rezucha and Reichard (2015) for the same trait. A longer time lag between courtship observations, compared with that of personality traits, was used to allow for the sperm and motivation level of all experimental fish to equate.

### 2.4 Emergence test

The emergence test measures boldness/exploration. An aquarium (40 × 20 × 30 cm) filled with 10 cm of water was used. This contained a dark, plastic box (10 × 10 × 10 cm) placed near one of the aquarium walls, which served as refuge. A blue or violet mat was placed under the aquarium, and the color was changed for each of the two replicates. Burns (2008) showed that an alternation in the test arena yields higher repeatability of the tests, probably by minimizing the effect of familiarization with the new environment in subsequent replicates. At the beginning of the trial, a male was put into the box through a hole cut in the ceiling, which was immediately re‐covered. After 5 min of acclimatization, the door in the front wall of the box was removed, which could be done discretely without being seen by the fish. Boldness was measured as the time taken by the male to emerge from the box (i.e., when his whole body was visible through the camera suspended above the aquarium). Males who emerged earlier into the open space of the unfamiliar aquarium were considered bolder. A maximum score of 300 s was assigned to those fish (14 individuals) that did not come out within 5 min of removing the door. Immediately after the trial, the fish were released back to the home aquarium to avoid familiarization with the test arena.

### 2.5 Open‐field test

An open‐field test was used to measure boldness and exploration. An aquarium (40 × 20 × 30 cm) filled with 10 cm of water was used, the walls of which were covered with opaque plastic to prevent distractions from outside the test area. The bottom was divided into 40, 5 × 5 cm squares. Each individual fish was gently released onto one of the central squares (the same for all males) and allowed to explore the unfamiliar aquarium for 4 min. Afterward, it was captured and put back into its home aquarium. Swimming rate, that is, the number of squares traversed when not frozen, was the measure of exploration. Fish with a higher score were considered more explorative. Time frozen (i.e., not moving) is interpreted as indicative of shyness–boldness (Burns, 2008). Males with higher scores were considered shyer, as freezing behavior resembles a natural reaction of guppies to the presence of predator (Templeton & Shriner, 2004).

### 2.6 Courtship behavior

Courtship behavior was observed when fish were situated in a 3‐L plastic container. At the beginning of the trial, the container was divided into two equal parts using a transparent plastic partition allowing for visual contact between individuals to be maintained. The focal male and a mature virgin outbred female from the stock population were placed separately into the two different compartments. Each female was only used once. After 5 min of acclimatization, the partition was removed and male behavior was recorded for 20 min. The number of sigmoid displays performed by each male was measured, as this is a typical courtship behavior for this species (Liley, 1966; Magurran & Seghers, 1990). A sigmoid display consists of S‐shape displays in front or to the side of the female. In the two cases where the male copulated with the female during the test, the rate of displays performed prior to the copulation was extrapolated to the remaining test time. Measurements are extrapolated in this way as male guppies exhibit a postcopulatory refractory period in which they do not display (Houde, 1997) and so the results could be biased.

### 2.7 Statistical analyses

#### 2.7.1 Test validity

Repeatability of courtship display, boldness, and exploration measures were calculated according to Lessells and Boag (1987), by dividing the among‐individual variance by the sum of the among‐ and within‐individual variances. Spearman correlation was used as a second measure of repeatability. Confidence intervals for repeatabilities, based on *F* ratios, were calculated following Nakagawa and Schielzeth (2010). Convergent validity of personality tests, that is, whether the tests measure the same trait, was calculated using Spearman correlations for all pairs of measures of personality traits (averages from block 1). Correlations between each of the personality measures (averages in case of repeated trials), and the number of sigmoid displays were calculated using Spearman method.

#### 2.7.2 Effect of inbreeding

As all three personality measures were significantly correlated (see Section 3), I applied a principal components analysis and then, using linear model, tested for the effect of treatment on the first principal component (PC1), which explained most variation and has an intuitive biological interpretation (see Sections 3 and 4). In this analysis, I included data from blocks 1 and 2 (averaged over the two trials), as in the last two blocks only one personality trait, latency to emerge, was measured. Block was fixed effect in this analysis, as there were only two of them, making estimation of error associated with random effect unreliable (Bolker et al., 2009).

Additionally, I tested the effect of treatment on each measured behavioral trait with a separate model. The effect of treatment on latency to emerge, on time frozen, and on courtship behavior was tested with negative binomial generalized linear mixed models for Poisson distribution of model residuals, while the effect on swimming rate was tested with a general linear mixed model. In all cases, treatment (inbred/outbred) was a fixed factor and male identity a random effect (to account for the repeated measurements in some blocks). Block was fixed, as there were too few of them to provide reliable estimation of error associated with random effect (Bolker et al., 2009).

Since I had a strong hypothesis regarding the effect of inbreeding on the number of sigmoid displays, I tested it with a directed test (Rice & Gaines, 1994). This incorporated critical regions for rejecting *H*_0_ in the anticipated and unanticipated direction set to 0.8 and 0.2, respectively, as recommended by Rice and Gaines (1994). All tests were performed in R 3.2.3 (R Core Team, 2015); specifically, stats and lme4 1.1.18 (Bates, Mächler, Bolker, & Walker, 2015) packages were used for testing the effect of treatment.

To estimate the magnitude of negative effect of inbreeding on the control trait–sigmoid display, I calculated the inbreeding depression coefficient, that is, the slope of change in trait values as a result of inbreeding, standardized by the outbred trait mean: bXo = (Xo − XI)/FXo (DeRose & Roff, 1999), where Xo is the mean trait value in outbreds, XI is the mean trait value in inbreds, and *F* is Wright's (Wright, 1921) inbreeding coefficient (0.25 in case of this study, that is, after one generation of brother–sister mating).

## 3 RESULTS

### 3.1 Tests validity

Repeatability (±CI) and correlation coefficients for all behavioral traits are given in Table 2. Time frozen was the only behavioral trait for which no significant correlation was present between the first and the second trial. However, the repeatability score was rather high. Results for convergent validity tests are given in Table 3. All measured traits were significantly correlated in the directions expected. None of the personality measures was significantly correlated with courtship behavior: latency to emerge: *r* = −.13, *p* = .19; swimming rate: *r* = .04, *p* = .65; freezing time: *r* = −.01, *p* = .88.

**Table 2:** Internal validity of behavioral tests

Trait	Spearman correlation		Repeatability	
	*r*_s_	*p*	*R*	95% CI
Latency to emerge	.63	**.000**	.64	0.60–0.68
Swimming rate	.43	**.001**	.60	0.40–0.80
Time frozen	.22	.123	.65	0.45–0.85
No of sigmoids	.56	**.003**	.29	0.022–0.38

Note

Spearman correlation coefficients together with its *p* values, and repeatabilities with confidence interval values. Significant *p* values are in bold.

**Table 3:** Convergent and discriminant validity of behavioral tests

Trait	Time frozen		Swimming rate	
	*r*_s_	*p*	*r*_s_	*p*
Latency to emerge	.27	**.005**	−.28	**.004**
Time frozen			−.58	**.000**

Note

Spearman correlation coefficients for all trait combinations, significant *p* values in bold. For calculating the correlation between time frozen and swimming rate, the data from the first minute of the open‐field test and the following 3 min were taken, respectively, in order to avoid nonindependence of data.

### 3.2 Effect of inbreeding

Principal components analysis resulted in three PC's, the first of which explained over half (58%) of the total variance in behavior. There was no difference in PC1 between treatments (*t*_1,110_ = 0.82, *p* = .41). (Full model is presented in Table 4; details on variance explained by the other two PC's and on loadings of individual measures of behavior are given in Table 5.)

**Table 4:** The models testing for the effect of treatment on PC1 of the principal components analysis, and on single traits measured, controlled for the effect of block

Behavioral trait	*N*	Explanatory variable	Estimate (*SE*)	Effect size^a^	*p*
PC1	108	Inbred	−0.19 (0.24)	−0.82	.41
		Block 2	1.00 (0.24)	4.28	**.00**
Swimming rate	109	Inbred	−0.06 (0.05)	1.10	.28
		Block 2	−0.14 (0.06)	−2.39	**.02**
Time frozen	109	Inbred	−0.35 (0.22)	−1.54	.12
		Block 2	0.49 (0.25)	2.00	**.05**
Latency to emerge	173	Inbred	0.16 (0.16)	−0.99	.32
		Block 2	0.47 (0.20)	2.30	**.02**
		Block 3	−0.24 (0.23)	−1.02	.31
		Block 4	0.01 (0.24)	0.03	.97
Number of sigmoids	165	Inbred	−0.29 (0.16)	−1.79	**.04**
		Block 2	−0.11 (0.20)	−0.54	.59
		Block 3	0.22 (0.24)	0.93	.35
		Block 4	0.21 (0.24)	0.90	.37

Note

*N* is the sample size. Significant *p*‐values are bolded.

a Effect size is expressed as *z* value, except for PC1 and swimming rate, where *t* values are reported.

**Table 5:** Loading values of individual behavior measures on the three PC's from the principal components analysis

	PC1 (0.58)	PC2 (0.25)	PC3 (0.16)
Latency to emerge	0.49	−0.87	−0.05
Swimming rate	−0.61	−0.39	0.69
Time frozen	0.62	0.31	0.72

Note

Proportion of total variance in behavior explained by each PC is given in parenthesis next to the PC label.

**Figure 1:**
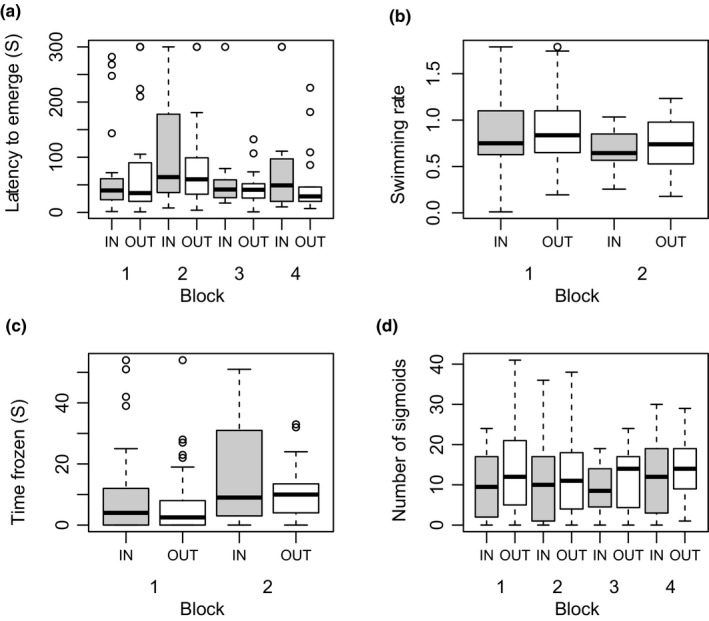
The effect of treatment on four behavioral measures: (a) latency to emerge from shelter; (b) swimming rate in open‐field trial; (c) time frozen during open‐field trial; and (d) number of sigmoid displays in the presence of a female. The boxes represent median ± interquartile range (IQR), whiskers denote min and max values (<1.5 IQR), and outliers are marked with open dots. Results for inbred (IN) and outbred (OUT) treatment are represented with gray and white boxes, respectively

None of the single personality trait measures was affected by the treatment: swimming rate: *t*_1,109_ = 1.10, *p* = .27; latency to emerge: *z*_1,173_ = 0.99, *p* = .40; time frozen: *z*_1,109_ = −1.54, *p* = .12. Sigmoid display, the control trait for the phenotypic effect of inbreeding, showed significant inbreeding depression (*t*_1,165_ = −1.80, *p* = .04; bXo = 0.79). Treatment effects are visualized in Figure 1. The effect of block was detected for swimming rate, emergence time, and time frozen. The interaction between treatment and block was not significant for either of the traits and was removed from the models. Details of final models are included in Table 4.

## 4 DISCUSSION

One of the hypotheses explaining consistent differences in personalities, that is, individually consistent behavioral traits, poses that the expression of costly behavioral traits is dependent on condition (Lewis, 2015; Luttbeg & Sih, 2010; Rands et al., 2003). Thus, individuals differing in condition will express different levels of personality traits. Here, I tested this hypothesis by manipulating condition with inbreeding. If the personality traits I investigated were condition‐dependent, I expected them to show the effects of inbreeding depression, similarly to what was reported for traits important for individual fitness (DeRose & Roff, 1999). Inbreeding, by negatively affecting condition, is also expected to decrease the values of condition‐dependent traits, resulting in their lower population mean. However, my results offer no evidence for such a scenario: Inbred males did not differ in personality trait levels relative to their outbred counterparts. The mean values of none of the measures of boldness and tendency to explore were affected by inbreeding, thus providing no indication that these traits are condition‐dependent (see Figure 1a–c). Also, none of the personality measures was significantly correlated with the number of sigmoid displays performed by a male, suggesting that the mechanism responsible for the effect of inbreeding on courtship behavior does not affect personality traits.

The results of the study were true both for individually tested behavior measures and for the first PC from the principal components analysis. The first PC explained over half of the total variance in behavior. Furthermore, it was the only of the three components that has an intuitive biological interpretation: It was positively loaded by latency to emerge and time frozen, while negatively by swimming rate. Thus, I interpret it as a combined measure of boldness and exploration, with bold and explorative individuals opposed to shy and less explorative ones. This is in line with the significant associations among the traits reported for the convergent and discriminant analyses.

The validity of the procedure was confirmed by the effect on courtship display. This trait was chosen as a control based on earlier reports on its susceptibility to inbreeding depression (Mariette et al., 2006; van Oosterhout et al., 2003). Here, sigmoid display was a significantly repeatable trait on an individual level, with outbred males performing significantly more sigmoid displays than their inbred counterparts (see Figure 1d, Table 4). The negative effect of inbreeding was associated with substantial inbreeding depression coefficient, 0.79, a value exceeding the mean for life‐history and morphological traits reported in a meta‐analysis of DeRose and Roff (1999). Nevertheless, the value of 0.79 is considerably lower than that described in a former study measuring inbreeding depression in the number of sigmoids (Mariette et al., 2006), possibly reflecting differences in the demographic history of the laboratory populations used in these studies, which may have resulted in their different inbreeding loads. It should be kept in mind that the inbreeding depression in sigmoid displays was only treated as evidence for the effectiveness of inbreeding manipulation, not as a reference for the strength of the effect of inbreeding. Personality traits and sexually selected traits (such as courtship display) may well experience different levels of inbreeding depression.

The lack of detectable effect of inbreeding on personality traits is unlikely to be due to insufficient level of inbreeding. Inbreeding coefficient increases in a logistic manner, and thus, the increase in inbreeding is the highest in the first inbred generation (see e.g., Falconer, 1989). Thus, a protocol used in this experiment, using one generation of inbreeding repeated in four blocks, thus allowing to measure large number of families, was more powerful in detecting inbreeding depression compared with a design where similar number of individuals would be tested across four generations of inbreeding. Despite this, in the present study there was no tendency observed in any direction for any of the measured traits.

The lack of detectable inbreeding depression is also unlikely to be due to insufficient repeatability of the tests used. Two out of three personality measures, that is, latency to emerge and swimming rate, showed high repeatabilities. These traits also showed significant correlations between the first and second trials, confirming that these tests are well suited for personality assays. The correlation between trials was not significant for time frozen. However, the repeatability calculated following Lessells and Boag (1987), which takes into account the number of replicates per individual, was reasonably high (*r* = .6). Additionally, time frozen was positively correlated with latency to emerge but negatively with swimming rate, that is, in the expected directions. Hence, although it should be treated with caution, time frozen seems to be an informative measure of personality. The 95% confidence intervals for repeatabilities were reasonably narrow for all behavior measures (see Section 3), and for all three personality measures, the lower value of repeatability was greater than the average level of 0.37 reported for those traits in a meta‐analysis by Bell, Hankison, and Laskowski (2009). As expected, latency to emerge and swimming rate were negatively correlated. The results of convergent validity tests are thus in line with those reported for the same study species by Burns (2008) in a study validating the open‐field and emergence tests. Open‐field test measures exploration and boldness, with swimming rate being more indicative of exploration tendency, whereas time frozen of boldness (Burns, 2008). In the emergence test, latency to emerge is considered the outcome of a conflict between shyness and propensity to explore (Burns, 2008). Thus, both tests are measuring different aspects from the shy–bold and explorative–nonexplorative continuum. There has been a concern about the validity of the emergence test as there is uncertainty as to whether guppies consider the dark shelter a safe refuge or not (see Burns, 2008). However, within this experiment, all guppies immediately hid in the shelter when the test was completed and I tried to recapture them with a net. This clearly indicates that the fish felt safer in the box. Taken together, the above validity tests give confidence that the result is not due to improper design of behavioral assays.

One explanation for the present result could be that personality traits do not affect fitness at all and are thus not costly, which would mean that their expression does not depend on condition. However, in the same laboratory population used in the present study, I found a positive association between males' boldness and their reproductive success (Herdegen‐Radwan *in preparation*) This is also in line with the results of studies on other guppy populations, which have revealed associations between sexually selected traits and boldness (Rezucha & Reichard, 2016), or female preference for bolder males (Godin & Dugatkin, 1996). Therefore, it seems unlikely that personality traits in the guppy could indeed be evolving neutrally.

It is worth mentioning that, although here I did not explore the genetic basis of personality traits, my results have implications for a hypothesis that specifically addresses the maintenance of genetic variation in personality traits, namely the mutation‐selection balance hypothesis. Mechanisms of condition dependence may be based solely on environmental factors, or they can have a genetic basis, because they likely constitute a large target for mutations (Rowe & Houle, 1996). In the latter case, genetic variation for condition might be maintained by a balance between mutation and selection. In such a case, even if selection is acting directionally, favouring extreme trait levels, genetic variation would not be depleted because of the continuous influx of deleterious mutations, which may segregate in populations for many generations if they are recessive or partially recessive and/or their effects are small (DeRose & Roff, 1999). This mutation load, according to the mutation‐selection hypothesis, is responsible for maintaining a range of different suboptimal trait values (Houle, 1998) and shifting the population mean of the trait away from its optimum. In the case of personality traits, this mechanism might operate either via their condition dependence or via the direct effect of deleterious mutations on personality traits, which are often highly polygenic and may constitute a large mutational target (Verweij et al., 2010). In either case, the effect of inbreeding, by revealing the load of recessive/partially recessive deleterious mutations, should affect personality traits. Thus, in addition to contradicting the condition‐dependence model of the maintenance of personalities, the results of the present study do not support the existence of a mutation‐selection balance that directly maintains variation in genes affecting personality traits.

As an alternative to both condition‐dependence and mutation‐selection balance, which were not supported by the result of the present study, balancing selective pressures may provide an explanation for the maintenance of variation in personality traits in guppy populations. Under such a scenario, no change in the mean trait value is expected under inbreeding, as there is no directional selection acting on it. Support for this mechanism has already been found in other species (Cote, Dreiss, & Clobert, 2008; Dingemanse & Reale, 2005). In guppies, Rezucha and Reichard (2016) showed that the intensity of courtship behavior is positively associated with boldness. This finding may suggest that bolder males could gain higher reproductive success, as females prefer courtship‐oriented mates over those that attempt coercive copulations (Kodric‐Brown & Nicoletto, 2001). This is consistent with what I found in the same population studied here (Herdegen‐Radwan *in preparation*), that is, that bolder males have higher reproductive success. If, at the same time, colorful, bold males have a higher predation risk, due to both conspicuous coloration and behavior, opposing selective forces may maintain differences in personalities. Further investigation of the ways in which personality traits affect male guppy survival should continue to shed light on this puzzle.

## CONFLICT OF INTEREST

The author declares that she has no conflict of interest.

## AUTHORS CONTRIBUTION

I am the only author of the manuscript.

## ETHICAL APPROVAL

All applicable international, national, and/or institutional guidelines for the care and use of animals were followed. The experimental protocols were approved by the Local Ethics Committee in Poznań (decision numbers 5/2015 from 14.4.2015 and 69/2017 from 19.01.2018). The breeding population is licensed and monitored by the veterinary inspectorate (license no PL30646224 of the Local Veterinary Inspectorate in Poznań). After the experiments, all inbred, experimental fish were kept under the conditions described above, in unisex groups, until their natural deaths. They were not reused in any further experiments. Outbred fish were returned to the stock population.

## Data Availability

Data on personality and courtship tests are deposited in Dryad (https://doi.org/10.5061/dryad.50d300k).
